# Molecular-Level Identification of Liquor Vintage via an Intelligent Electronic Tongue Integrated with a One-Dimensional Convolutional Neural Network

**DOI:** 10.3390/s25237350

**Published:** 2025-12-03

**Authors:** Yali Bi, Yalong Zhu, Jiaming Liu, Digan Yu, Qiqing Fan, Xuefeng Hu, Wei Zhang

**Affiliations:** 1 Anhui Province Key Laboratory of Measuring Theory and Precision Instrument, School of Instrument Science and Optoelectronics Engineering, Hefei University of Technology, Hefei 230009, China; ylbi@hfut.edu.cn (Y.B.); zaaron794@gmail.com (Y.Z.); nerveva93@163.com (J.L.); 2024110015@mail.hfut.edu.cn (D.Y.); 2024110044@mail.hfut.edu.cn (Q.F.); xuefeng.hu@hfut.edu.cn (X.H.); 2 College of Integrated Circuit Science and Engineering, Nanjing University of Posts and Telecommunications, Nanjing 210023, China

**Keywords:** electronic tongue, one-dimensional convolutional neural network (1D-CNN), machine learning, deep learning, liquor vintage identification, electrochemical sensing, molecular recognition

## Abstract

Accurate identification of liquor vintage is crucial for ensuring product authenticity and optimizing market value, as the price and sensory quality of liquor increase with age. Traditional sensory evaluation by sommeliers is inherently limited by subjectivity, physiological fatigue, and inconsistency, posing challenges for reliable large-scale quality assessment. To address these limitations, this study introduces an innovative homemade electronic tongue (ET) system integrated with machine learning and deep learning algorithms for rapid and precise vintage identification. The ET system, consisting of six metallic electrodes and a MEMS-based temperature sensor, successfully discriminated five consecutive liquor vintages produced at one-year intervals. Using Support Vector Machine (SVM) and Random Forest (RF) algorithms, classification accuracies of 91.0% and 78.0% were achieved, respectively. Remarkably, the proposed one-dimensional convolutional neural network (1D-CNN) model further improved the recognition accuracy to 94.0%, representing the highest reported performance for ET-based vintage prediction to date. The findings demonstrate that the integration of multi-electrode electrochemical sensing with artificial intelligence enables objective, reproducible, and high-throughput evaluation of liquor aging characteristics. This approach provides a scientifically robust alternative to human sensory analysis, offering significant potential for counterfeit detection, liquor authentication, and the broader assessment of food and beverage quality within molecular sensing frameworks.

## 1. Introduction

Liquor represents a predominant category within the global liquor industry, occupying a central position in both production and consumption markets. Newly produced liquor is typically characterized by turbidity, reduced clarity, and an astringent, acidic taste profile that reflects its chemical immaturity. To achieve desirable organoleptic and compositional stability, liquor must undergo a controlled period of storage and aging during which a series of physicochemical transformations—including redox reactions, esterification, condensation, and polymerization—occur, progressively enhancing its structural and sensory attributes [1]. These maturation processes are fundamental to the evolution of aroma complexity, color stabilization, and flavor harmonization that define high-quality liquor. Aged liquor consistently exhibits superior sensory and flavor characteristics relative to its younger counterparts, features that are essential for attracting consumers and sustaining the long-term equity of established brands. Among the multiple factors influencing liquor quality and market perception, the vintage—the year indicated on the label corresponding to the grape harvest—remains of paramount importance, as it encapsulates the combined effects of climatic conditions, viticultural practices, and grape biochemical composition, all of which decisively shape fermentation dynamics and aging outcomes. Consequently, vintage serves not only as a critical indicator of liquor authenticity and quality but also as a major determinant of market valuation, exerting a substantial influence on liquorry profitability and brand positioning within the global marketplace.

Traditional liquor aging has long relied on the use of oak barrels, a practice that imparts structural complexity and distinctive aromatic characteristics through gradual oxygen exchange and wood compound extraction. In addition to barrel maturation, several other conventional techniques—such as aging on lees (AOL), temperature-controlled aging, and biological aging—have been employed for centuries to refine liquor quality and stability [2]. With the advancement of enological science, a range of artificial or accelerated aging technologies has emerged to replicate or enhance the natural maturation process while significantly reducing production time. These include the application of oak adjuncts and derivatives [3]; controlled chemical micro-oxygenation methods that simulate slow oxidative evolution [4]; and various physical acceleration techniques, such as ultra-high-pressure treatment [5], pulsed electric fields [6], ultrasonic irradiation [7], microwave exposure [8], and other irradiation-based processes [9]. More recently, coupled or hybrid technologies integrating multiple physical and chemical mechanisms have been developed to further optimize reaction kinetics and improve flavor development efficiency [10]. Despite these technological innovations, the predominant industrial practice for producing aged or vintage-style liquors remains the blending of base liquors with components of different maturation stages or vintages, allowing producers to achieve a targeted balance of sensory and chemical attributes [11].

Given the high economic value and strong consumer demand associated with branded liquors, the global market has increasingly faced challenges related to product authenticity. A major concern is the proliferation of counterfeit and substandard liquors, including those falsely labeled with fabricated or misleading vintages [12]. In recent years, the problem of alcohol adulteration and counterfeiting has escalated sharply, driven by complex distribution networks and inadequate traceability mechanisms. Such fraudulent products not only compromise consumer trust but also pose serious risks to public health due to potential contamination and uncontrolled chemical compositions. Moreover, the widespread circulation of counterfeit liquors disrupts market stability, undermines brand integrity, and causes substantial economic losses to legitimate producers [13]. Consequently, the development of rapid, accurate, and reliable analytical technologies for verifying liquor authenticity and determining vintage has become a critical priority. These advanced detection methods are indispensable for ensuring product safety, safeguarding consumer confidence, and maintaining the orderly operation of the global liquor market.

The traditional reliance on professional sommeliers for evaluating liquor flavor and identifying counterfeit or inferior products presents inherent limitations in meeting modern analytical and quality assurance requirements. Human sensory evaluation, while valuable for descriptive profiling, lacks the capacity to ensure consistency, reliability, and reproducibility across large-scale detection tasks. The judgment of sommeliers can be influenced by numerous uncontrollable external and psychological factors—including individual taste preferences, consumption habits, cultural background, and even transient emotional states—which introduce substantial variability into the assessment process [14]. Such subjectivity inevitably leads to inconsistencies and potential bias, thereby reducing the accuracy and objectivity of liquor classification and authenticity verification. Consequently, the development of instrument-based analytical approaches capable of performing precise, stable, and repeatable identification of liquor quality has become indispensable. These technologies not only overcome human limitations but also provide standardized, data-driven frameworks for objective evaluation and counterfeit detection in the liquor industry.

Currently, instrumental methods employed for liquor quality classification primarily include gas chromatography–mass spectrometry (GC–MS) [15], ultra-high-performance liquid chromatography (UHPLC) [16], high-performance liquid chromatography–mass spectrometry (HPLC–MS) [17,18], and ultraviolet–visible (UV–Vis) spectrophotometry [19]. These analytical techniques are recognized for their exceptional accuracy and sensitivity in detecting and characterizing volatile and non-volatile compounds that contribute to liquor quality and authenticity. However, their practical application is constrained by the need for large-scale laboratory instrumentation, high operational costs, and complex sample preparation procedures, which collectively limit their accessibility for routine or on-site analysis. In recent years, the development of miniaturized and rapid detection technologies—including infrared spectroscopy [20], electrolyte-free nanogap electrochemical cells [21], and piezoelectric sensing systems [22]—has opened new avenues for efficient liquor identification. These emerging techniques significantly reduce measurement complexity and analysis time, thereby enhancing portability and field applicability. Nonetheless, they often exhibit comparatively lower analytical sensitivity and accuracy than conventional chromatographic and spectrometric platforms. Therefore, the development of a simple, rapid, accurate, and efficient analytical method for reliable liquor quality identification remains a critical objective in contemporary enological research and quality assurance practice.

As a bionic detection technology, the electronic tongue (ET) is designed to simulate the sensory perception mechanisms of the human gustatory system and offers the advantages of rapid, accurate, non-invasive, and adaptable analysis across diverse application contexts [23,24]. The ET functions by capturing the physicochemical and biochemical signal responses of liquid samples through an array of taste sensors, which collectively generate multidimensional data reflecting the overall composition of the sample. Subsequent data processing and pattern recognition are achieved through computational modeling, typically involving multivariate statistical analysis, machine learning, or deep learning algorithms to classify and interpret complex signal patterns [25]. Recent studies have demonstrated the ET’s capability in discriminating among various sample types, including different varieties of apple juice [26], botanical origins of honey [27], degrees of water pollution [28], and the aging of Chinese rice liquor [29], as well as in detecting counterfeit honey [30] and distinguishing between animal-derived food products such as pork [31], tomatoes [32], and milk [33]. Moreover, several investigations have confirmed the feasibility of using ET systems to classify liquors by age or vintage with promising accuracy [34,35,36,37,38,39,40]. These applications collectively highlight the technical potential of electronic tongue technology in liquor authentication, particularly for vintage determination and counterfeit liquor identification. Nevertheless, despite its demonstrated versatility, current ET systems still require optimization in terms of sensitivity, accuracy, and model generalization to fully meet the stringent analytical standards of industrial-scale liquor quality evaluation [41,42].

Recent advances in artificial intelligence (AI) and machine learning (ML) algorithms have considerably enhanced the capacity for liquor quality assessment and vintage prediction. For instance, Yu et al. [43] integrated Least Squares Support Vector Machines (LS-SVM) with near-infrared (NIR) spectral data to predict and classify rice liquor ages, demonstrating that LS-SVM achieved superior predictive performance compared with traditional discriminant analysis (DA) methods. However, the relatively limited sample size (147 liquor labels) constrained the model’s generalization ability, leading to suboptimal prediction accuracy. Similarly, Astray et al. [44] explored alternative AI-based regression and classification techniques—including Artificial Neural Networks (ANN), Support Vector Machines (SVM), and Random Forest (RF) models—to enhance age prediction precision. Their findings indicated that the RF algorithm exhibited robust predictive capacity even under small-sample conditions (<60 samples), yielding improved Root Mean Squared Error (RMSE) and Average Absolute Percentage Deviation (AAPD) metrics. In another approach, Abbal et al. [45] developed a Bayesian network–based decision-support system that achieved an overall classification accuracy of approximately 75%, further demonstrating the promise of probabilistic reasoning in enological prediction tasks. Collectively, these studies establish a foundational framework for applying AI-driven algorithms to liquor age estimation. Nevertheless, despite encouraging progress, the predictive accuracy and robustness of current models remain insufficient for consistent implementation in industrial liquor quality control and large-scale authentication practices.

In this study, we report for the first time the application of an electronic tongue (ET) for the identification of liquor vintage with a one-year age resolution. The objective of this study is to discriminate the production year of a single specific liquor brand across multiple vintages. A custom-built voltammetric electronic tongue (VE tongue) was developed, incorporating six metallic working electrodes—gold (Au), silver (Ag), platinum (Pt), palladium (Pd), tungsten (W), and titanium (Ti)—in combination with a MEMS-integrated temperature sensor to ensure precise environmental control during measurement. The system operated using multi-frequency large-amplitude pulse voltammetry (MLAPV) scanning waveforms, enabling the acquisition of rich electrochemical response profiles. Due to the differing electrochemical sensitivities of the metallic electrodes, each generated distinct response characteristics, collectively providing multidimensional discriminatory information about the liquor samples. To enhance analytical accuracy, a one-dimensional convolutional neural network (1D-CNN) algorithm was embedded within the intelligent ET framework for automated feature extraction and classification of liquor vintages. Compared with conventional machine learning classifiers, the 1D-CNN model demonstrated superior performance in distinguishing subtle aging differences among liquor samples. The system achieved a classification accuracy of 94.0% in discriminating liquor vintages within a one-year interval, markedly outperforming traditional machine learning–based identification methods and underscoring the potential of deep-learning-assisted ET systems for rapid, accurate, and intelligent liquor authentication.

## 2. Results

### 2.1. Electronic Tongue Signals

**Figure 1:**
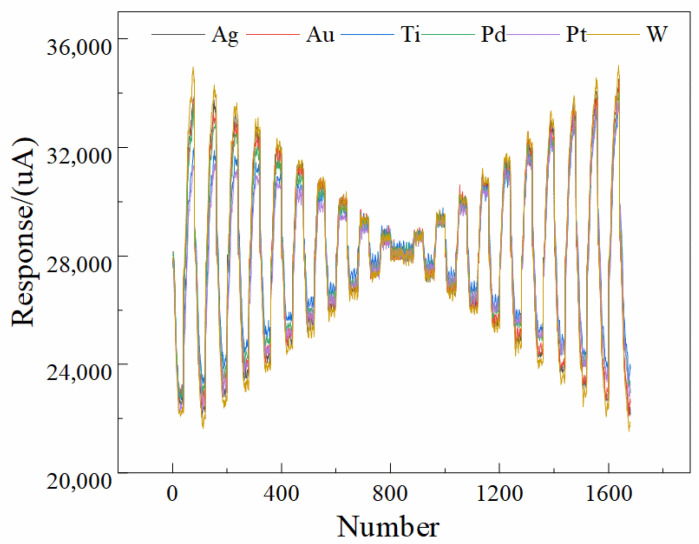
Representative current response curves of liquor samples obtained using the homemade electronic tongue (ET) system with six metallic electrodes. Each curve exhibits distinct electrochemical fingerprint characteristics corresponding to different liquor vintages, reflecting the unique redox behavior and compositional variations among samples.

The raw current response signals acquired by the six working electrodes of the electronic tongue system are presented in Figure 1. Each sample generated a total of 10,080 data points (6 electrodes × 1680 sampling points per electrode). The response curves clearly reveal that each electrode exhibited distinct electrochemical behavior, forming unique “fingerprint” patterns corresponding to liquor samples of different vintages [46,47,48]. This observation indicates that the six metallic electrodes possess differential sensitivities toward various physicochemical constituents—such as organic acids, phenolic compounds, and lipid derivatives—present in liquors of different aging years.

The variation in electrode response may arise from the diverse electrochemical redox mechanisms occurring at the surfaces of different metal electrodes [49,50,51]. These redox interactions reflect compositional changes in the liquor matrix associated with aging, particularly the transformation of active components such as lipids and organic acids over time [52]. It is well established that phenolic compounds in liquor undergo oxidation at the electrode surface, while acids (e.g., formic acid) and esters (e.g., alkyl esters) are reduced at the cathode, thereby generating measurable current responses [53]. The differences in current intensity and waveform shape across electrodes thus provide multidimensional electrochemical information that serves as the analytical basis for vintage classification and subsequent feature extraction.

**Figure 2:**
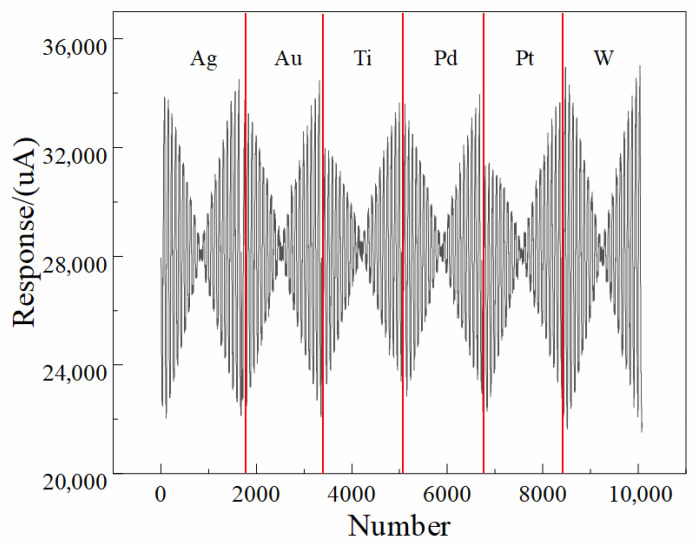
Input signal representation of the one-dimensional convolutional neural network (1D-CNN) model. The feature matrix is constructed from the multi-electrode response data acquired by the electronic tongue (ET) system, serving as the neural network’s input for automated feature extraction and classification of liquor vintages.

Each voltage plateau generated 40 evenly spaced sampling points, resulting in a total of 42 × 40 = 1680 data points per electrode per measurement. These 1680 points therefore represent the time-resolved current response associated with 42 excitation pulses, rather than a single linear voltammetric scan, and collectively form the multi-channel input matrix used in Figure 1 and the 1D-CNN model described in the abstract and conclusion. Each electrode measures 1680 response data to a single sample, so the response data of the six electrodes necessitate to be processed horizontally and then exercised as the input of the network, as shown in Figure 2.

### 2.2. SVM Recognition

**Figure 3:**
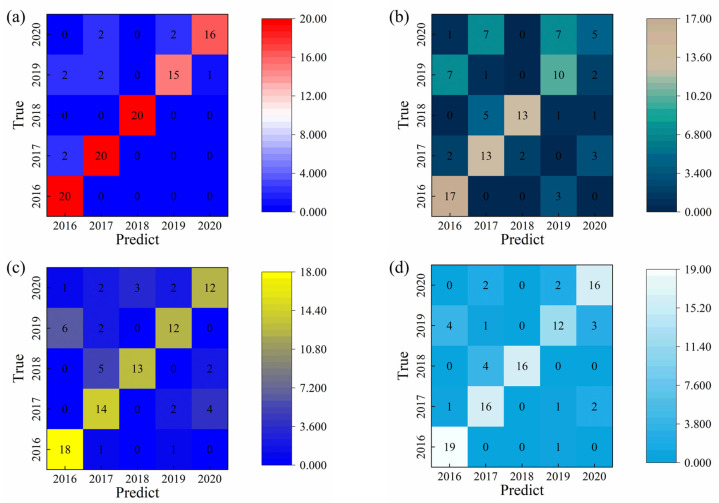
Support Vector Machine (SVM) classification performance using different feature extraction approaches: (**a**) classification based on integrated area values; (**b**) classification based on maximum response values; (**c**) classification based on minimum response values; and (**d**) classification using the original unprocessed data. The comparison demonstrates the influence of feature selection on model accuracy and robustness in liquor vintage identification.

The Support Vector Machine (SVM) model was optimized by systematically tuning its key hyperparameters—the kernel function, penalty coefficient (C), and kernel parameter (γ)—using a grid search strategy to achieve the best classification performance. The optimization process indicated that the linear kernel function, combined with a penalty parameter of C = 6, yielded the most effective configuration for the dataset. Under these conditions, the integral area feature was identified as the most discriminative variable for model training and classification. Using the optimized parameters, the SVM model achieved a test set classification accuracy of approximately 91.0%, demonstrating strong performance in distinguishing liquor vintages based on the extracted electrochemical features. The classification accuracies of individual liquor components obtained after parameter optimization are summarized in Table 1, while the confusion matrix illustrating the detailed classification distribution across different vintages is presented in Figure 3. These results confirm that the SVM model, when appropriately tuned, can effectively capture the linear separability characteristics of the extracted features for accurate vintage identification.

### 2.3. RF Identification

**Figure 4:**
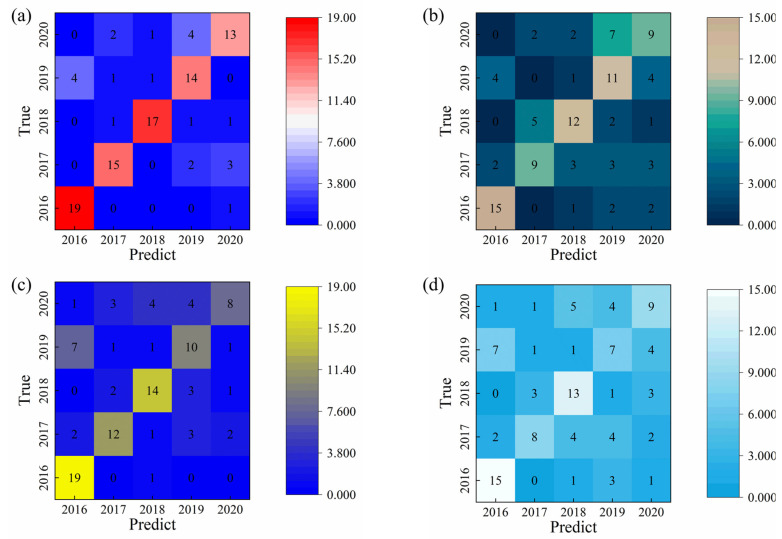
Random Forest (RF) classification performance based on different feature extraction methods: (**a**) classification using integrated area values; (**b**) classification using maximum response values; (**c**) classification using minimum response values; and (**d**) classification using the original unprocessed data.

For the Random Forest (RF) model, key hyperparameters—including the number of features considered at each split (max_features), the maximum tree depth (max_depth), and the number of decision trees (n_estimators)—were optimized using a grid search algorithm to enhance model performance. The optimal configuration was obtained when max_features = 8, max_depth = 8, and n_estimators = 64. Under these conditions, the RF classifier achieved a test set accuracy of 78.0% when using the integral area values as the primary eigenfeatures. The classification accuracy corresponding to each feature variable after model optimization is summarized in Table 2, and the confusion matrix depicting the distribution of classification outcomes across different liquor vintages is shown in Figure 4. Although the RF algorithm exhibited reliable classification capability, its overall performance was lower than that of the SVM model, likely due to the relatively limited feature dimensionality and moderate inter-class variability among the liquor samples. Nonetheless, the RF model provided valuable comparative insight into the effectiveness of ensemble-based classifiers for liquor vintage identification.

### 2.4. 1D-CNN Recognition

**Figure 5:**
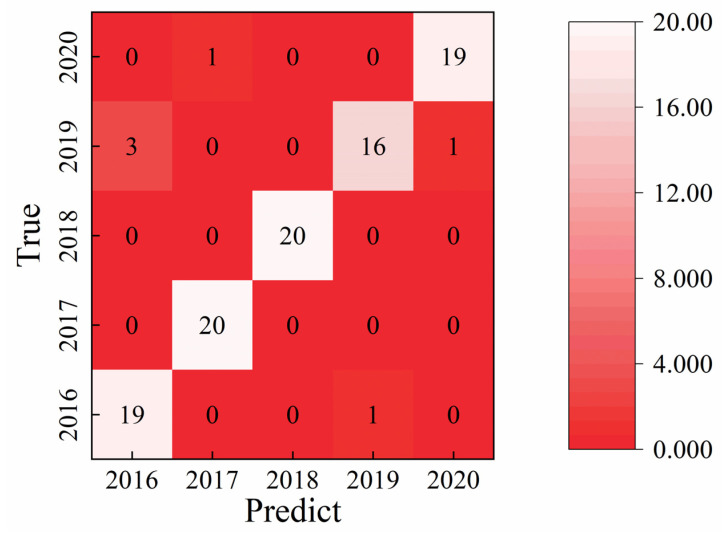
Classification performance of the one-dimensional convolutional neural network (1D-CNN) model for liquor vintage identification. The deep learning model achieves a recognition accuracy of 94.0%, demonstrating superior feature extraction and classification capability compared with traditional machine learning algorithms.

The one-dimensional convolutional neural network (1D-CNN) model parameters were systematically optimized to achieve optimal classification performance. The optimization process focused on key factors including the network architecture, optimizer type, batch size, and number of training iterations. The final optimized configuration parameters are summarized in Table 3. After 100 training epochs, the model exhibited convergence and stability, indicating effective learning of the electrochemical signal features. As illustrated in Figure 5, the optimized 1D-CNN model achieved a test set classification accuracy of 94.0%, with only six samples misclassified across all vintage categories. This result demonstrates the strong discriminative capability and robustness of the 1D-CNN approach in identifying subtle compositional differences among liquor samples of adjacent aging years. Compared with traditional machine learning models such as SVM and RF, the deep learning-based 1D-CNN achieved a substantially higher recognition accuracy, confirming its superior ability to extract nonlinear and hierarchical features from complex electrochemical data for precise liquor vintage classification.

## 3. Discussion

The non-zero and nearly identical baseline current across all six electrodes does not represent a true Faradaic current; rather, it results from the intrinsic characteristics of the two-electrode configuration used in our electronic tongue (ET) system, as well as the measurement electronics and circuit design. Specifically, because the system employs a polarizable stainless-steel counter electrode and lacks an internal non-polarizable reference electrode, the recorded “zero-potential” condition corresponds to the externally applied potential difference between the working array and the counter electrode, but does not ensure that each individual working electrode is at its own equilibrium open-circuit potential. Under this configuration, slight residual potential offsets, input bias currents from the potentiated circuitry, and the inherent polarization of the stainless-steel counter electrode contribute to a stable but non-zero measured current even when the nominal applied potential is zero. In addition, the multi-channel acquisition electronics utilize synchronized sampling circuitry that introduces a small, constant bias current associated with the analog front-end amplifier, which appears as a uniform baseline across all electrodes regardless of their material compositions. Because this offset is governed predominantly by the instrument electronics and is identical for all channels after calibration and filtering, it produces the nearly uniform ~28 mA baseline observed in the figures. Importantly, this baseline current does not affect the discriminative power of the ET, because the machine learning and deep learning pipelines operate on the dynamic, potential-dependent components of the signal rather than the global offset. The waveform features that distinguish liquor vintages arise from electrode-dependent changes in impedance, ion mobility, interfacial polarization, oxide behavior, and adsorption interactions, all of which manifest as variations in slope, curvature, and transient behavior across the voltammetric waveform rather than the fixed baseline. Figure 2 further illustrates that the 1D-CNN model processes normalized multichannel signals; therefore, constant offsets are effectively treated as non-informative and do not influence feature extraction.

The use of a polarizable stainless-steel counter electrode inevitably alters the electrochemical response characteristics and that this configuration differs fundamentally from a classical three-electrode system in which the working-electrode potential is precisely controlled with respect to a non-polarizable reference electrode. The current–potential behavior shown in Figure 1 and the multi-frequency pulse waveform of Figure 2 indeed exhibit a predominantly linear relationship between current amplitude and applied voltage. This observation indicates that the measured signals contain a substantial ohmic component dominated by the combined resistance of the liquor matrix, the electrode interfaces, and the passivating oxide films formed on the metallic electrodes, rather than representing purely Faradaic oxidation–reduction reactions. The ET signals should not be interpreted as direct measurements of the intrinsic redox kinetics of specific electroactive species. Instead, the system captures a composite electrochemical fingerprint that reflects variations in matrix conductivity, interfacial impedance, adsorption characteristics, capacitive charging behavior, and polarization dynamics—all of which are influenced by subtle physicochemical differences among liquor vintages. Although the current response is predominantly ohmic, the heterogeneity of the six metallic electrodes introduces electrode-specific modulation of resistance, interfacial charge distribution, oxide growth behavior, and ion transport phenomena, which collectively produce reproducible multichannel patterns that encode the compositional, structural, and aging-related differences among samples. These multidimensional waveform features, rather than isolated Faradaic peaks, constitute the information extracted by the machine-learning and deep-learning models described, explaining why the 1D-CNN achieved the highest recognition accuracy (94.0%) by capturing nonlinear correlations within the composite time-series signal. In the revised text. The two-electrode design was chosen to achieve a compact, reference-free, integrated platform suitable for automated, high-throughput liquor analysis, consistent with the engineering and application-driven objectives emphasized in conclusion. It clearly differentiate between electrochemical mechanisms that are directly redox-controlled and those dominated by electrolyte/electrode resistance and polarization, and we have corrected any statements implying that the measured current arises exclusively from Faradaic processes. Instead, the observed signals constitute rich impedance-like fingerprints whose discriminatory power arises from the complex interplay of material-dependent polarization characteristics under the MLAPV excitation shown in Figure 2.

**Figure 6:**
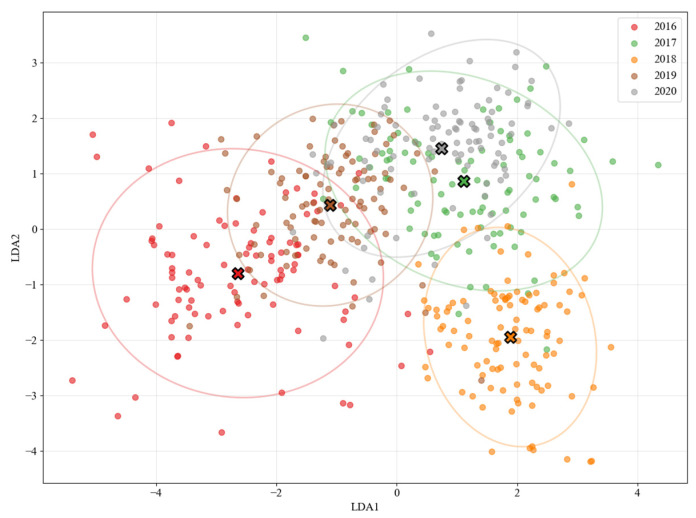
Linear Discriminant Analysis (LDA) results for liquor samples of five production years (2016–2020). Sample points are color-coded by year. multiplication signs are the centroids.

The dimensionality-reduced results of the LDA are shown in Figure 6. The ‘X’ markers represent class centroids, and the ellipses indicate 95% confidence intervals. The plot clearly visualizes the separation among liquor samples from 2016 to 2020: the 2016 (red), 2017 (green), and 2018 (yellow) clusters are well separated, while the 2019 (brown) and 2020 (orange) samples exhibit partial overlap and are closer to the 2018 cluster, suggesting a higher degree of compositional similarity among these vintages.” We also added the following sentence in Section 3: “The classification results of the Random Forest model are consistent with the visual distribution observed in the LDA plot, in which the 2019 and 2020 samples are located closer to the remaining groups.

**Figure 7:**
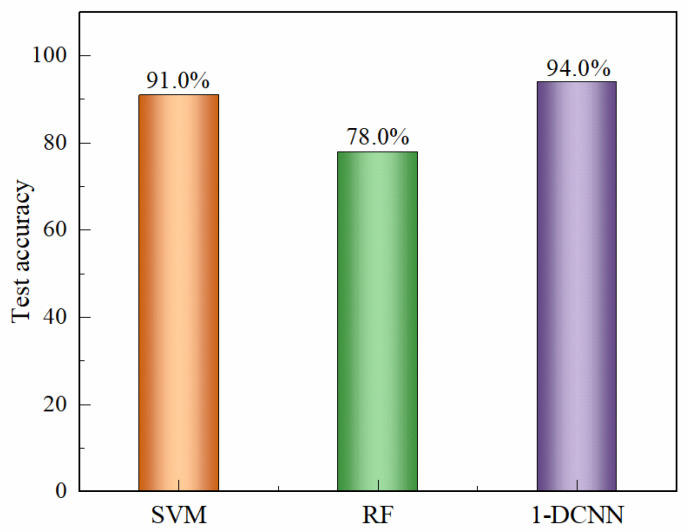
Comparison of recognition accuracies obtained using different classification algorithms, including Support Vector Machine (SVM), Random Forest (RF), and one-dimensional convolutional neural network (1D-CNN). The 1D-CNN model demonstrates the highest classification performance, achieving an accuracy of 94.0%, outperforming traditional machine learning approaches in identifying liquor vintages.

Figure 7 summarizes the comparative recognition accuracies of liquor vintage classification obtained using the SVM, RF, and 1D-CNN algorithms. The limitation and to explain that LDA, RF, SVM, and CNN analyses collectively provide a multi-method confirmation of the vintage-discriminative patterns. Among the traditional machine learning approaches, the SVM model achieved the highest test set accuracy of 91.0%, whereas the RF model reached 78.0% when the integral area features were used as input variables. The classification results of the Random Forest model are consistent with the visual distribution observed in the LDA plot, in which the 2019 and 2020 samples are located closer to the remaining groups. In contrast, the 1D-CNN model developed in this study demonstrated a test set accuracy of 94.0%, surpassing the SVM by approximately 3.0%. The superior performance of the 1D-CNN can be attributed to its capacity to automatically extract nonlinear and hierarchical features from the raw electrochemical data, thereby capturing subtle compositional and temporal variations across liquor samples of different vintages. These results clearly illustrate that deep learning architectures, particularly CNN-based models, offer a more powerful and generalizable framework for accurate liquor vintage identification compared with conventional machine learning methods.

The superior identification accuracy of liquor vintages achieved by the 1D-CNN model can be attributed to its integrated dual functionality of feature extraction and classification, as well as its ability to utilize the complete temporal information captured by the electronic tongue system. Unlike traditional machine learning algorithms that rely solely on manually engineered features, the 1D-CNN autonomously learns multilevel feature representations directly from the raw signal data. The use of convolution kernels of varying sizes and quantities enables the model to extract deep hierarchical features, capturing both local and global signal characteristics that are critical for accurate classification. In contrast, conventional machine learning methods possess only classification capabilities and depend on manual feature extraction processes, which often limit their representational capacity. Moreover, these models typically learn only low-level statistical features from the input data [54], thereby neglecting complex nonlinear patterns and inter-feature dependencies. This limitation leads to reduced recognition accuracy and weaker generalization performance compared with deep learning-based architectures.

## 4. Material and Methods

### 4.1. Sample Collections

The liquor samples used in this study were provided by Golden Seeds Liquor Company (Hefei, China), a well-known producer of premium liquors. The samples consisted of five consecutive vintages—2016, 2017, 2018, 2019, and 2020—representing blended high-grade liquors from the same production line to ensure batch consistency. The beverages analyzed in this study were not red or white grape wines; instead, all samples were Chinese distilled liquors (baijiu). The liquors used across the five vintages contained an alcohol content of 52% (*v*/*v*), consistent with their commercial labeling. For each production year, all samples were taken from a single bottle of liquor. For each vintage, 100 individual samples were collected, yielding a total of 500 liquor specimens for analysis. All samples were stored under identical conditions prior to testing to minimize the influence of environmental variability. The detailed distribution of the liquor samples by vintage is presented in Table 4.

### 4.2. Electronic Tongue (ET) System

**Figure 8:**
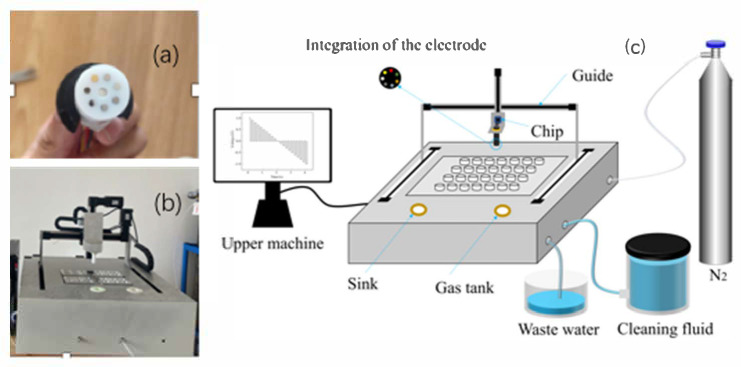
Schematic representation of the homemade intelligent electronic tongue (ET) system: (**a**) integrated multi-electrode sensor array comprising six noble metallic electrodes (Ag, Au, Ti, Pd, Pt, and W); (**b**) complete configuration of the homemade ET system integrating signal acquisition, temperature control, and automated cleaning modules; (**c**) operational schematic of the ET testing system illustrating the signal generation, data acquisition, and analytical workflow.

The configuration of the homemade electronic tongue (ET) system, comprising both hardware and software components, is illustrated in Figure 8. In general, the materials most frequently employed for the fabrication of ET sensor arrays include metals, carbon-based materials, polymers, metal oxide semiconductors, and macromolecular compounds [46]. Among these, metallic electrodes have garnered particular attention due to several distinct advantages: (1) their straightforward preparation and reproducibility; (2) the wide diversity in the physicochemical properties of different metals, which facilitates selective electrode construction and enhances the system’s overall discrimination capability; and (3) the inherent chemical and physical stability of most noble metals, which are resistant to corrosion and unlikely to react with the test matrix.

In this study, the ET sensor array was constructed using six metallic electrodes—silver (Ag), gold (Au), titanium (Ti), palladium (Pd), platinum (Pt), and tungsten (W)—each serving as an individual sensing element to generate a multidimensional electrochemical response profile. The geometric working area of each metallic sensing electrode was approximately 1 mm^2^, defined by the exposed circular surface of the electrode embedded in the PTFE matrix. The overall design principle of our ET system is to construct a heterogeneous and complementary sensor array capable of generating rich, multidimensional electrochemical fingerprints that reflect subtle compositional variations among liquor vintages. The metals with distinct standard electrode potentials, catalytic activities, oxide-formation tendencies, surface adsorption characteristics, and electron-transfer kinetics were intentionally selected. Ag provides high sensitivity to anions and organic acids through the formation of Ag^+^/AgOx species; Au offers stable adsorption-controlled responses toward polyphenols and sulfur-containing compounds; Ti and W form passivating oxide layers (TiO_2_ and WO_x_) that contribute capacitive and ion-intercalation behavior sensitive to pH and matrix ionic strength; Pd exhibits characteristic hydrogen/oxygen adsorption and alcohol/aldehyde oxidation responses; and Pt serves as a broad-spectrum electrocatalytic probe for redox-active organic constituents. Although some electrodes, such as Ag or Pd, may undergo partial dissolution or surface oxide formation at anodic potentials up to +1 V. It is confirmed through pre-conditioning, automated cleaning, and temperature stabilization that these processes were highly reproducible across all measurements and thus contributed consistent discriminatory information rather than interfering noise. The diversity of electrochemical processes across the six electrodes intentionally enhances the dimensionality and complexity of the recorded voltammetric curves, which directly benefits machine-learning analysis.

A micro-platinum temperature sensor was integrated into the electrode array to enable in situ temperature monitoring and maintain measurement stability. Each electrode was embedded in a polytetrafluoroethylene (PTFE) matrix, which provided electrical insulation, physical separation, and precise spatial alignment between the sensing units. The PTFE block also acted as a mechanical support and formed part of the microfluidic chamber through which the liquor samples flowed during automated loading and measurement, preventing unintended electrical cross-talk between electrodes. The stainless-steel tube served as the counter electrode, and the system operated without a reference electrode, simplifying the overall configuration while maintaining reliable performance. During measurement, the applied potential pulses enabled the ET system to extract and record the characteristic electrochemical “fingerprint” signals of the liquor samples, which were subsequently processed for pattern recognition and classification.

The STM32F03C8T6 microcontroller served as the core processing unit of the ET signal acquisition circuit. Operating at a frequency of 72 MHz, the STM32F03C8T6 integrates a 12-bit analog-to-digital converter (ADC), 512 KB of Flash memory, and 160 KB of RAM, enabling efficient communication with the host computer and real-time control of all peripheral functional modules. Signal acquisition and waveform generation were achieved through an AD5941 (Analog Devices, Wilmington, MA, USA) 16-bit data acquisition unit, which offers a channel sampling rate of up to 800 kS/s. This module incorporates an integrated digital waveform generator and digital filtering system, which together facilitate the generation and conditioning of precise excitation signals.

**Figure 9:**
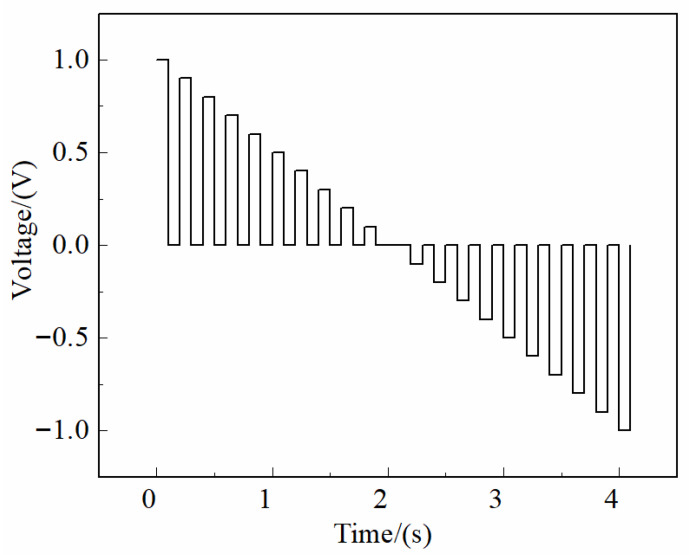
Applied pulse voltage sequences and a representative square-wave current response obtained from the metallic working electrode. The waveform illustrates the multi-frequency large-amplitude pulse voltammetry (MLAPV) excitation pattern used for electrochemical sensing, where each pulse segment induces distinct redox processes, generating characteristic current responses corresponding to the liquor samples.

As illustrated in Figure 9, the excitation waveform was a trapezoidal voltage pulse with an amplitude range of −1 V to +1 V, a step increment of 0.1 V, and a peak and trough duration of 0.1 s for each square segment. Following each pulse sequence, a 0 V holding potential was applied for 2 s to ensure complete electrode desorption and signal stabilization. This waveform design effectively balances excitation strength and temporal resolution, ensuring sensitive electrochemical response capture. The system was capable of acquiring stable, high-fidelity current signals from all six working electrodes simultaneously, thereby providing a robust and reliable electrochemical dataset for subsequent pattern recognition and classification analysis.

The constant-temperature control module employed a TEC1 semiconductor thermoelectric refrigeration chip, enabling rapid heating and cooling through bidirectional current regulation. Temperature modulation was achieved by adjusting both the magnitude and polarity of the applied current. To ensure precise thermal regulation, the integrated Pt100 temperature sensor embedded within the electrode array continuously monitored the sample temperature in real time. The temperature data were transmitted to the host computer to achieve closed-loop feedback control, thereby maintaining highly stable thermal conditions during measurement. This high-precision temperature regulation system ensured that all detection procedures were conducted under constant-temperature conditions, minimizing thermal drift and improving data repeatability.

The driving electrode module was composed of an XYZ three-axis linear positioning system coupled with a stepper motor driver, allowing automated spatial movement of the electrodes. Through the host computer interface, the stepper motors executed precise three-dimensional motion in accordance with pre-programmed protocols, enabling automated sampling and measurement operations. The sample-cleaning module included a water tank, inlet and outlet pipelines, an air tank, a water pump, and an electromagnetic relay. Prior to and following each measurement cycle, the electrode array was automatically positioned above the cleaning tank, where the computer-controlled water pump and relay sequentially activated to perform washing and purging procedures. This fully automated cleaning system effectively removed residual substances and prevented cross-contamination between samples, thereby ensuring measurement integrity and system longevity.

### 4.3. Experimental Procedure

All measurements were conducted under controlled temperature conditions at 25 ± 1 °C to ensure consistency and minimize thermal interference. Prior to each test, the electrode array was positioned above the cleaning tank and automatically subjected to a washing cycle to remove any residual contaminants. Following cleaning, approximately 7 mL of the selected liquor sample was transferred into the sample tank. The host computer then programmed the driving module to precisely align the electrode array with the sample position, allowing the electrodes to make contact with the liquid surface.

During measurement, the electrodes were excited using a pulse voltage waveform generated by the built-in voltage source, initiating the voltammetric scanning process. The resulting electrochemical response signals were converted into digital binary data via the data acquisition card and transmitted in real time to the host computer interface for storage and subsequent signal processing and pattern analysis. After each measurement cycle, the electrodes were sequentially rinsed with deionized water and anhydrous ethanol for 15 s to remove any adhering residues, followed by high-pressure nitrogen purging for 15 s to ensure complete air-drying before subsequent use. This standardized cleaning and drying protocol effectively prevented cross-contamination and maintained measurement reproducibility across all experimental runs.

### 4.4. Data Preprocessing

The original data set of the experiment is considered 500 × 10,080. The difference between the measured current response peaks of each sample is too significant, leading to very complicated calculations and affecting the convergence speed of the model. In this way, the normalization method [47] requires to preprocess the original data, as shown in Formula (1),

(1) $$X_{scaler}=\frac{X-X_{min}}{X_{max}-X_{min}}$$

where $X_{scaler}$, X, $X_{min}$, and $X_{max}$ are the normalized data, the original response data, the minimum value of each feature of the initial response data, and the maximum value of each component of the actual response data, respectively.

### 4.5. Feature Signal Extraction

Feature signal extraction is a fundamental process in machine learning that aims to remove redundant or non-informative variables from the original dataset, thereby reducing computational complexity and mitigating the risk of model overfitting. Effective feature extraction plays a critical role in constructing robust and generalizable classification models [48]. In this study, feature extraction was performed on the raw electrochemical response data obtained from the electronic tongue system. Specifically, for each response waveform, three quantitative descriptors were derived: (1) the integral area corresponding to 20 consecutive response data points surrounding the abscissa following each square pulse; (2) the maximum response value; and (3) the minimum response value. These selected features collectively represent the dynamic and steady-state characteristics of the electrode response, serving as the input variables for subsequent machine learning and deep learning analyses.

### 4.6. Machine Learning

Two machine learning (ML) algorithms were employed in this study: Support Vector Machine (SVM) [49] and Random Forest (RF) [50]. The SVM is a kernel-based supervised learning model widely applied in classification and regression tasks involving relatively small and high-dimensional datasets. It functions by constructing an optimal hyperplane that maximizes the margin between different classes, thereby improving generalization performance. In contrast, the RF algorithm operates as an ensemble learning method, integrating the results of multiple decision trees to form a robust composite classifier. Each individual tree performs an independent classification, and the final decision is obtained through majority voting among all trees. This ensemble mechanism effectively reduces overfitting and enhances model stability. Due to its capability to manage complex, nonlinear relationships and noisy datasets, RF has demonstrated high classification accuracy across a wide range of real-world applications. Both algorithms were implemented for comparative analysis to evaluate their effectiveness in predicting and classifying liquor vintages based on the extracted feature signals.

### 4.7. Deep Learning

The Convolutional Neural Network (CNN) is a widely applied deep learning architecture composed of convolutional layers, pooling layers, and fully connected layers [51]. CNNs are trained through an iterative optimization process in which model parameters are updated by minimizing the loss function using gradient descent combined with the chain rule and backpropagation algorithms. This approach allows CNNs to automatically extract hierarchical features from raw input data, thereby achieving high accuracy, strong generalization ability, and operational stability across complex datasets.

The one-dimensional convolution operation, built-in in this study, is similar to the traditional multiple-dimensional convolution. It assumes that the input sequence is x and the number of convolution kernels w is k. Convolve x and then add a bias b, which activates a nonlinear function to the output feature. The process of one-dimensional convolution expresses as the following formula:

(2) $$x^{k}=f(x\times w^{k}+b^{k})$$

where f(⋅) is the nonlinear activation function.

The activation function is responsible for the nonlinear weighting of the convolutional features, moderating the linearity of the entire network and advancing the mapping ability of the network. Comparing the two activation functions of Sigmoid and Tanh, when the input of the Relu activation function is less than 0, the output is directly 0, which has a specific light effect and can prevent overfitting from a certain extent the training process. When the input is greater than 0, the function’s inverse is 1, and there is no gradient vanishing problem. Therefore, the ReLU function for each convolution stage’s activation function and expresses as follow:

(3) f(x)=max(0,x)

The pooling layer is used as a sub-sampling layer and has no parameters for training. Its rule is to employ a pooling window to ‘scan’ the adjacent parameter values at a particular position of the input feature map to obtain the overall statistical features as the input of the next layer of the network.

In the whole network framework, flattening is adopted at the latest pooling stage, enabling the extraction of multi-dimensional feature mapping into one-dimensionalities. In addition, flatten inserts a Dropout layer, which can randomly simply parameters, thereby avoiding the occurrence of overfitting and ensuring computational efficiency in the stage of training the model.

The last layer of the model uses five neurons and the Softmax function for probability normalization to output five label category results.

(4) $$softmax=\frac{e^{x_{i}}}{\sum_{i}^{n}e^{x_{i}}}$$

**Figure 10:**
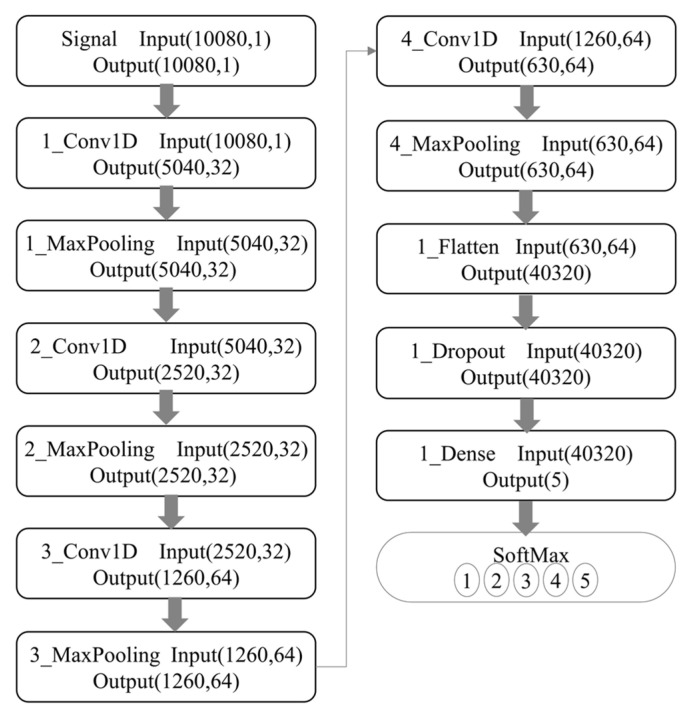
1-DCNN architecture flow.

The liquor sample response data measured by the intelligent electronic tongue system is a multi-channel one-dimensional vector, so building a one-dimensional convolutional neural network (1D-CNN) classification task model is essential. Figure 10 shows the network structure used in the experiment. The first convolutional layer uses 32 convolution kernels of size 16 × 1; the second, 32 convolution kernels of size 8 × 1; the third, 64 convolution kernels of size 4 × 1, and the fourth, 64 convolution kernels of size 2 × 1. The stride of all convolution kernels is 2, and the activation function is ReLU. Max-pooling performers after each convolution, and all max-pooling layers are of size 2 × 1 and stride of 1. After the convolution, the flat layer, discarding, and the final Softmax classifier identifies five different vintages.

The model’s training and test sets account for 0.8 and 0.2 of the total samples, respectively. The recognition program is written and implemented on a Dell 3681 computer (Windows10 system, Intel i5 processor, 8G running memory, Pycharm2019 software, configuration environment Python3.7, Scikit-learn0.21.3, tensorflow2.1.0, keras2.3.1).

## 5. Conclusions

In this study, a homemade high-performance electronic tongue (ET) system was successfully designed and applied for the precise identification of liquor vintages. The system integrates six metallic electrodes—silver, gold, titanium, palladium, platinum, and tungsten—together with a MEMS-based temperature sensor, forming a compact, reference-electrode-free detection platform capable of automatic temperature regulation, sample loading, cleaning, data acquisition, and signal analysis. This configuration enables high-throughput, multi-channel, and low-abundance detection, substantially reducing manual operation and improving efficiency. By coupling the ET system with a one-dimensional convolutional neural network (1D-CNN), the model achieved a classification accuracy of 94.0% in distinguishing liquor vintages within a one-year interval, representing, to the best of our knowledge, a state-of-the-art level of precision for ET-based vintage recognition [34,35,36,37,38,39,40]. The remarkable performance of the 1D-CNN model arises from its integrated capability for both feature extraction and classification, allowing it to capture complex nonlinear relationships within the raw electrochemical signals that traditional machine learning algorithms cannot effectively exploit.

Looking forward, future research should focus on improving the robustness, generalization, and interpretability of the ET–CNN framework and exploring its broader applicability in other analytical domains. More advanced neural network architectures, such as recurrent neural networks (RNNs), long short-term memory (LSTM) models, or transformer-based time-series frameworks, could be investigated to better capture temporal dependencies and dynamic response patterns in electrochemical signals. The integration of feature fusion strategies combining ET data with complementary sensing modalities, such as electronic nose (EN), infrared spectroscopy, or Raman spectroscopy, may provide multidimensional chemical fingerprints that enhance classification accuracy and system reliability. Additionally, incorporating cross-validation methods and Bayesian or genetic optimization algorithms for hyperparameter tuning would improve model stability and minimize overfitting. Emphasis should also be placed on developing explainable AI approaches—for example, using SHAP or Grad-CAM—to visualize and interpret learned features, thereby enhancing transparency and trust in industrial deployment. On the hardware side, efforts should be directed toward miniaturization and portability by integrating microcontrollers and wireless data transmission modules (e.g., Wi-Fi, Bluetooth, or IoT interfaces) to facilitate real-time, on-site vintage monitoring. Moreover, expanding the dataset to include a wider range of liquor varieties, geographical origins, and fermentation conditions would increase the model’s universality and robustness. Beyond the enological field, the proposed ET–CNN architecture holds great promise for cross-industry applications, including the quality control of fermented foods (such as soy sauce, vinegar, and traditional liquors), adulteration detection in agricultural and food products, and chemical and environmental analyses involving biomass materials, soil trace metals, or aqueous solutions. Through these continued advancements in deep learning, sensor fusion, and system integration, the ET-based intelligent sensing platform is expected to evolve into a universal, rapid, and accurate analytical tool for large-scale quality inspection, authenticity verification, and counterfeit detection across multiple scientific and industrial domains.

## Figures and Tables

**Table 1:** SVM classified results.

Feature	Test Accuracy
AREA	91.0%
MAX	58.0%
MIN	69.0%
ORIGINAL	79.0%

**Table 2:** RF classified results.

Feature	Test Accuracy
AREA	78.0%
MAX	56.0%
MIN	63.0%
ORIGINAL	52.0%

**Table 3:** The detailed 1D-CNN parameters.

Item	Parameter
Dropout	0.3
Optimizer	Adam
Lr	0.001
Loss	Categorical cross-entropy
Batch size	32
Epoch	100

**Table 4:** Sample information.

Category	Number of Samples	Train Set	Test Set
2016	100	0.8	0.2
2017	100	0.8	0.2
2018	100	0.8	0.2
2019	100	0.8	0.2
2020	100	0.8	0.2

## Data Availability

The data that support the findings of this study are available from the corresponding author upon reasonable request.
